# Microglia from neurogenic and non-neurogenic regions display differential proliferative potential and neuroblast support

**DOI:** 10.3389/fncel.2014.00180

**Published:** 2014-07-16

**Authors:** Gregory P. Marshall, Loic P. Deleyrolle, Brent A. Reynolds, Dennis A. Steindler, Eric D. Laywell

**Affiliations:** ^1^Departments of Anatomy and Cell Biology, College of Medicine, University of FloridaGainesville, FL, USA; ^2^Department of Neurosurgery, College of Medicine, University of FloridaGainesville, FL, USA; ^3^Department of Biomedical Sciences, College of Medicine, Florida State UniversityTallahassee, FL, USA

**Keywords:** microglia, inducible neurogenesis, proliferation, functional heterogeneity, subependymal zone

## Abstract

Microglia isolated from the neurogenic subependymal zone (SEZ) and hippocampus (HC) are capable of massive *in vitro* population expansion that is not possible with microglia isolated from non-neurogenic regions. We asked if this regional heterogeneity in microglial proliferative capacity is cell intrinsic, or is conferred by interaction with respective neurogenic or non-neurogenic niches. By combining SEZ and cerebral cortex (CTX) primary tissue dissociates to generate heterospatial cultures, we find that exposure to the SEZ environment does not enhance CTX microglia expansion; however, the CTX environment exerts a suppressive effect on SEZ microglia expansion. Furthermore, addition of purified donor SEZ microglia to either CTX- or SEZ-derived cultures suppresses the expansion of host microglia, while the addition of donor CTX microglia enhances the over-all microglia yield. These data suggest that SEZ and CTX microglia possess intrinsic, spatially restricted characteristics that are independent of their *in vitro* environment, and that they represent unique and functionally distinct populations. Finally, we determined that the repeated supplementation of neurogenic SEZ cultures with expanded SEZ microglia allows for sustained levels of inducible neurogenesis, provided that the ratio of microglia to total cells remains within a fairly narrow range.

## Introduction

Microglia are the resident immune cells of the CNS, and are located diffusely throughout the parenchyma. Under normal conditions microglia are deemed to be in a state of interactive surveillance but are rapidly activated by trauma, ischemia, infection, neuronal death, neoplasia, and degeneration (reviewed by Streit, 2002). Activation results in increased microglia number as a consequence of both the proliferation of resident cells, and the recruitment of circulating hematopoietic cells that infiltrate the CNS (Ajami et al., 2007; Lambertsen et al., 2011). The primary role of microglia has classically been described as that of sentinels responsible for maintaining brain homeostasis in the face of neurological insult (Schwartz et al., 2006). Activated microglia phagocytose cellular debris, such as degenerating neurons (Thanos, 1991; Huizinga et al., 2012; Suzumura, 2013), and they express complement receptors and act as cytotoxic effector cells (Gehrmann et al., 1995).

In addition to the “reactive” or “defensive” roles for microglia, evidence continues to increase suggesting that microglia also play vital roles in modulating normal neural function. A number of published reports indicate that microglia regulate the migration, proliferation, and differentiation of neural stem/progenitor cells (Battista et al., 2006; Butovsky et al., 2006; Walton et al., 2006). Microglia are also implicated in regulating synaptic density both on regenerating motor neurons following peripheral nerve lesion (Blinzinger and Kreutzberg, 1968; Trapp et al., 2007) and during neural development (Paolicelli et al., 2011). There is also compelling evidence that microglia and associated immune factors modulate neurogenesis both *in vivo* and *in vitro*. For instance, using an environmental enrichment paradigm known to augment adult hippocampal neurogenesis, Ziv et al. (2006) showed microglia activation to be associated with increased neuronal production. These authors also reported that Severe Combined Immunodeficiency (SCID) mice display impaired levels of neurogenesis under normal conditions, and that these levels are not responsive to environmental enrichment. Using an adrenalectomy model, Battista et al. (2006) were able to correlate increased hippocampal neurogenesis with the number of activated microglia in the dentate gyrus. Microglia have also been reported to play a crucial role in a unique form of inducible neurogenesis (IN) specific to mixed glial cultures derived from the subependymal zone. In this paradigm the withdrawal of serum and mitogens triggers massive de novo neuron production. The presence of microglia has been shown to be require for IN, and their loss -due either to serial passaging or selected immuno-ablation- results in a concomitant decline in the capacity for IN (Walton et al., 2006).

Microglia have also traditionally been viewed as functionally homogeneous throughout the neuraxis. Antecedent *in vitro* experiments typically have not involved the comparison of function by microglia from finely dissected anatomical regions, but rather use gross CNS areas (e.g., “forebrain”) to generate microglial cultures (e.g., Giulian and Baker, 1985). Recent data, however, are beginning to reveal additional functional roles for microglia (Walton et al., 2006), and to uncover substantial spatial heterogeneity in microglia function. For example, Goings et al. (2006) demonstrated that microglia in the adult SEZ are constitutively activated (as evidenced by their semi-amoeboid morphology and high density of CD45 and CD11b), in comparison to microglia in other regions. In addition, they showed that the SEZ contains more proliferating microglia than other areas, and that SEZ microglia respond uniquely to a variety of brain insults, theorized to be due in part to their being in a “primed” state of semi-activation. It has been reported that microglia are capable of performing both pro-neurogenic and anti-neurogenic roles in the injured brain, and that these dichotomous functions are temporally regulated. For example, endogenous microglia in the immediate vicinity of neurological insult become activated and begin to phagocytose dead cells while also secreting a variety of inflammatory chemokines such as tumor necrosis factor- α (TNF-α), interferon-γ (IFN-γ) and interleukin-1β, all of which are believed to play a role in the suppression of neurogenesis (reviewed in Ekdahl et al., 2009). However, in the weeks following injury, elevated numbers of insulin-like growth factor-1 (IGF-1) secreting microglia proliferate and accumulate in the SEZ, potentially playing a neuro-supportive role by promoting neuroblast migration to the site of injury. Finally, the secretion of TNF-α and IFN-γ by the insult-adjacent microglia plays a critical but indirect role in the induction of neighboring astrocytes to secrete ciliary neurotrophic factor, which supports re-myelination of neurons as well as neuronal survival (Simard and Rivest, 2004). Finally, we have previously reported that microglia from the SEZ are capable of massively greater population expansion than microglia from non-neurogenic brain regions (Marshall et al., 2008), suggesting a link between microglia proliferation and neurogenesis, and suggesting possible environmental factors within the neurogenic niche that promote or preserve proliferative capacity.

In the present study we ask two major questions. First, is the capacity for massive *in vitro* population expansion an intrinsic property of neurogenic zone microglia, or is it conferred by interactions within the neurogenic niche? Second, can *in vitro* neurogenesis be enhanced by manipulating the number of neurogenic zone microglia?

## Materials and methods

### Animals

All cultures were derived from neonatal (2–3 days post-birth) C57BL/6 wild type (WT) mice or C57BL/6 mice homozygous for the green fluorescent protein (GFP) reporter gene (Hadjantonakis et al., 1998). Animals were housed at the University of Florida's Department of Animal Care Services in compliance with IACUC regulations.

### Generation of region-specific cultures

**Figure 1:**
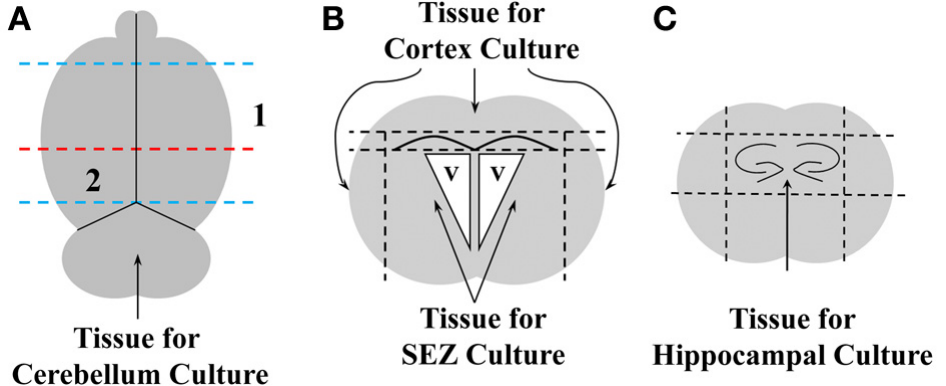
**Schematic representation of brain dissections**. The dorsal surface of whole brain was viewed **(A)**, and a razor blade was used to remove the olfactory bulbs and the hindbrain (blue dashed lines). The remaining tissue was bisected through the frontal plane (dashed red line), and areas 1 and 2 were further dissected to obtain cerebral cortex (CTX), subependymal zone (SEZ), and dorsal hippocampus (HC). For dissection of CTX and SEZ, area 1 was viewed in the coronal **(B)**, and dorsal and lateral cortical tissue superficial to the subcortical white matter was removed with razor cuts (upper horizontal dashed line & vertical dashed lines). SEZ was then obtained by first removing the subcortical white matter (lower dashed horizontal line in panel B), then finely dissecting the tissue immediately subjacent to the lateral ventricles (v). Finally, HC was obtained by viewing area 2 in the coronal plane **(C)** and blocking the tissue around the two dentate gyri by making razor cuts indicated by the four dashed lines.

Primary neural tissue was obtained from the brains of either WT or GFP+ neonatal mice, as schematized in Figure 1. Brains were dissected into discrete regions by first removing the olfactory bulbs and cerebellum with a sterile razor blade (blue lines in Figure 1A). The brain was then blocked by a coronal cut just anterior to the hippocampal formation (red line in Figure 1A) to generate fractions containing the SEZ and cerebral cortex (CTX, Numeral 1, Figure 1A), or hippocampus (Numeral 2, Figure 1A). The CTX was then separated from the SEZ by removing the tissue dorsal to the corpus callosum and lateral to the lateral ventricles (Figure 1B). The SEZ was further isolated by removing and discarding the white matter superior to the lateral ventricles. Finally, the hippocampus was isolated by removing the tissue surrounding the dentate gyri (dashed lines in Figure 1C). All tissue blocks were then separately minced with a sterile scalpel, and incubated for 15 min in ice-cold Dulbecco's Modified Eagles Medium (DMEM)/F12 media w/ HEPES and L-Glutamine (Gibco BRL, Carlsbad, CA; 11330-032) supplemented with antibiotic and anti-mycotic agents (Penicillin-Streptomycin, Gibco, 15140-122, and Fungizone Antimycotic, Gibco, 15295-017). The tissue was then pelleted by centrifugation at 400×g for 5 min, and incubated in 0.25% Trypsin/EDTA solution (Atlanta Biologicals, Atlanta, GA; B81310) at 37°C for 5 min. Trypsin activity was quenched by the addition of 1/5 volume fetal bovine serum (FBS: Atlanta Biological), after which a single-cell slurry was prepared by repeated trituration through a series of descending diameter fire-polished glass Pasteur pipettes. The slurry was washed in 5X volume DMEM/F12 and pelleted as above. Cells were re-suspended in neural growth medium (NGM) consisting of DMEM/F12 containing 5% FBS, N2 supplement (Gibco BRL, 17502-048), 20 ng/mL recombinant human epidermal growth factor (EGF; rhEGF, Sigma-Aldrich, St. Louis, MO; E9644), and 10 ng/mL basic fibroblast growth factor (FGF; bFGF, Sigma-Aldrich, F0291), plated onto tissue culture T-25 flasks and incubated for 3 days at 37°C in 5% CO2. Confluent cultures were trypsinized and re-plated at a 1:3 density into T-75 tissue culture flasks in NGM (passage 1). Passage 1 cultures were supplemented every 2–3 days with EGF/FGF until confluency was reached. The passaging procedure was then repeated as described above. At each passage a small aliquot of cells was placed onto poly-ornithine (P-Orn) coated glass coverslips and fixed for immunophenotyping (see below).

### Flow cytometric analysis of cell cycle and Mcm2

Confluent passage-5 monolayer cultures were incubated in the presence of the thymidine analog EdU (Click-iT® EdU Alexa Fluor® 488 Flow Cytometry Assay Kit, Invitrogen, Carlsbad, California; Cat. No. C35002) for 18 h. Cells were then trypsinized, collected, fixed and labeled for EdU according to manufacturer's recommendation. Cells were subsequently immunolabeled with anti-CD11b primary antibody directly conjugated to phycoerythrin (BD Pharmingen, San Jose, California; Catalog number 553311), as well as with the kit-supplied cell cycle label (633-red). Controls were prepared by generating each of the following from each culture group: unstained negative control; EdU signal only; cell cycle only; and CD11b-conjugated to phycoerythrin (PE) only. Samples were processed using a BD LSRII flow cytometer (BD Biosciences, San Jose, California) utilizing FACSDiva software. Unstained and IgG stained control cells were used to determine compensation parameters sufficient for diminishing fluorochrome signal overlap and producing clearly defined labeled-populations. Data were analyzed by FlowJo software (Treestar, Ashland, Oregon).

Mini chromosome maintenance protein 2 (Mcm2) in microglia was determined by first trypsinizing, collecting and fixing confluent SEZ and CTX monolayers at passage 3, 4, and 5. Cells were then co-immunolabled with rabbit anti-Mcm2 primary antibody (D7G11, Cell Signaling Technologies, Danvers, MA) as well as the anti-CD11b primary antibody directly conjugated to phycoerythrin used above. Following washing to remove residual primary antibody, secondary staining to label the bound Mcm2 antibody was conducted using FITC-conjugated Goat anti-Rabbit secondary antibody (BD Pharmingen, Catalog Number 554020). Stained cells were washed to remove residual secondary antibody then resuspended in flow cytometry buffer consisting of phosphate-buffered saline (PBS) with 10% fetal bovine serum (FBS) and 1% Sodium Azide). Samples were processed using a BD LSRII flow cytometer (BD Biosciences, San Jose, California) utilizing FACSDiva software using unstained, primary Mcm2 antibody alone and IgG stained control cells to determine compensation parameters sufficient for diminishing fluorochrome signal overlap and producing clearly defined labeled-populations.

### Expansion and isolation of microglia from adherent cultures

Microglia were expanded and isolated from adherent cultures as previously reported (Marshall et al., 2008). Briefly, NGM was replaced with microglial proliferation medium (MPM) consisting of DMEM/F12, 10% FBS, N2 supplement, and 20 ng/mL recombinant mouse granulocyte macrophage-colony stimulating factor (rGM-CSF, Stem Cell Technologies Inc; #02735). After 3 days in MPM, a microglia shake-off was performed by agitating the culture flasks for 10 minutes at room temperature (RT) on a rotary shaker set for 100 r.p.m. Detached cells was collected and quantified, while fresh MPM was added to the parent culture allowing for subsequent shake-offs at 3-day intervals. Isolated microglia were subsequently used as “donors” to supplement established mixed glial cultures (described below).

### Inducible neurogenesis in adherent SEZ cultures

Adherent WT SEZ cultures were established on P- Orn coated glass coverslips. Inducible neurogenesis (IN) was initiated upon confluence by serum starvation and mitogen withdrawal (Scheffler et al., 2005). The NGM was removed and replaced with DMEM/F12 media containing N2 supplement, but without serum or growth factors. Forty-eight hours after induction, small, phase-bright neuroblasts are abundant in the adherent cultures. Both induced and non-induced control cultures were fixed with 4% paraformaldehyde at 48 h. post- induction, and processed for immunolabeling as described below.

### Immunolabeling

All analyses were performed on confluent cultures, except for isolated microglia that were plated at low density and allowed to attach to coverslips for 24 h. Cells were fixed by immersion in PBS containing 4% paraformaldehyde (15 min @ RT), then washed with PBS for 5 min and blocked at RT for 1 h in PBS plus 0.01% Triton X-100 (PBSt) and 10% fetal bovine serum (FBS). Primary antibodies were applied to the cells overnight with gentle agitation at 4°C in PBSt containing 10% FBS. Primary antibodies: rat monoclonal anti-CD11b/Mac-1 (Becton Dickinson, Franklin Lakes, NJ: 550282, dilution 1:250) to label microglia; and rabbit polyclonal anti-β-III Tubulin (Covance; Princeton, NJ; PRB-435P, dilution 1:5000) to label neurons. Residual primary antibody was removed by 3 × 5 min washes with PBS. Secondary antibodies were applied at RT for 50 min in PBSt plus 10% FBS. Secondary antibodies were either goat anti-rat IgG conjugated to Alexa Fluor 568 (Molecular Probes, Eugene, OR: A11077), or goat anti-rabbit IgG conjugated to rhodamine (Molecular Probes, R-6394). Unbound secondary antibodies were removed by 3 × 5 min washes in PBS. Cells were coverslipped in Vectashield mounting medium containing the nuclear counterstain, DAPI (Burlingame, CA: H-1200).

### Microscopy and quantitative analysis

Cells were analyzed using a Leica DM2500 upright microscope (Leica Microsystems AG, Wetzlar, Germany) equipped with epifluorescence and aMagnafire digital camera (Optronics®, Goleta, California). Quantification was performed by capturing random fields of view at 20× magnification using DAPI filter cube to identify cell nuclei. The same field of view was photographed using first Leica I3 (GFP) filter followed by the TX2 filter (rhodamine). Merged images were then processed using ImageJ to determine the percent contribution of GFP and Rhodamine labeled cells to the total cell number. The values from 5 fields-of-view were combined and averaged to generate an estimate of the total cellular distribution within each culture. Additonal images for photodocumentation were captured using the Olympus DSU-IX81 (Olympus, Center Valley, PA) spinning disc confocal microscope.

### Statistical analysis

Quantitative values obtained from ImageJ were entered into Excel spreadsheets for graphical presentation, and into Graphpad Prism 4 for statistical analysis using either one- or Two-Way ANOVA with a Bonferroni *post-hoc* test of significance (significance was defined as a *p*-value ≤ 0.05).

## Results

### Neurogenic zone microglia have more proliferative cell cycle kinetics than CTX microglia

**Figure 2:**
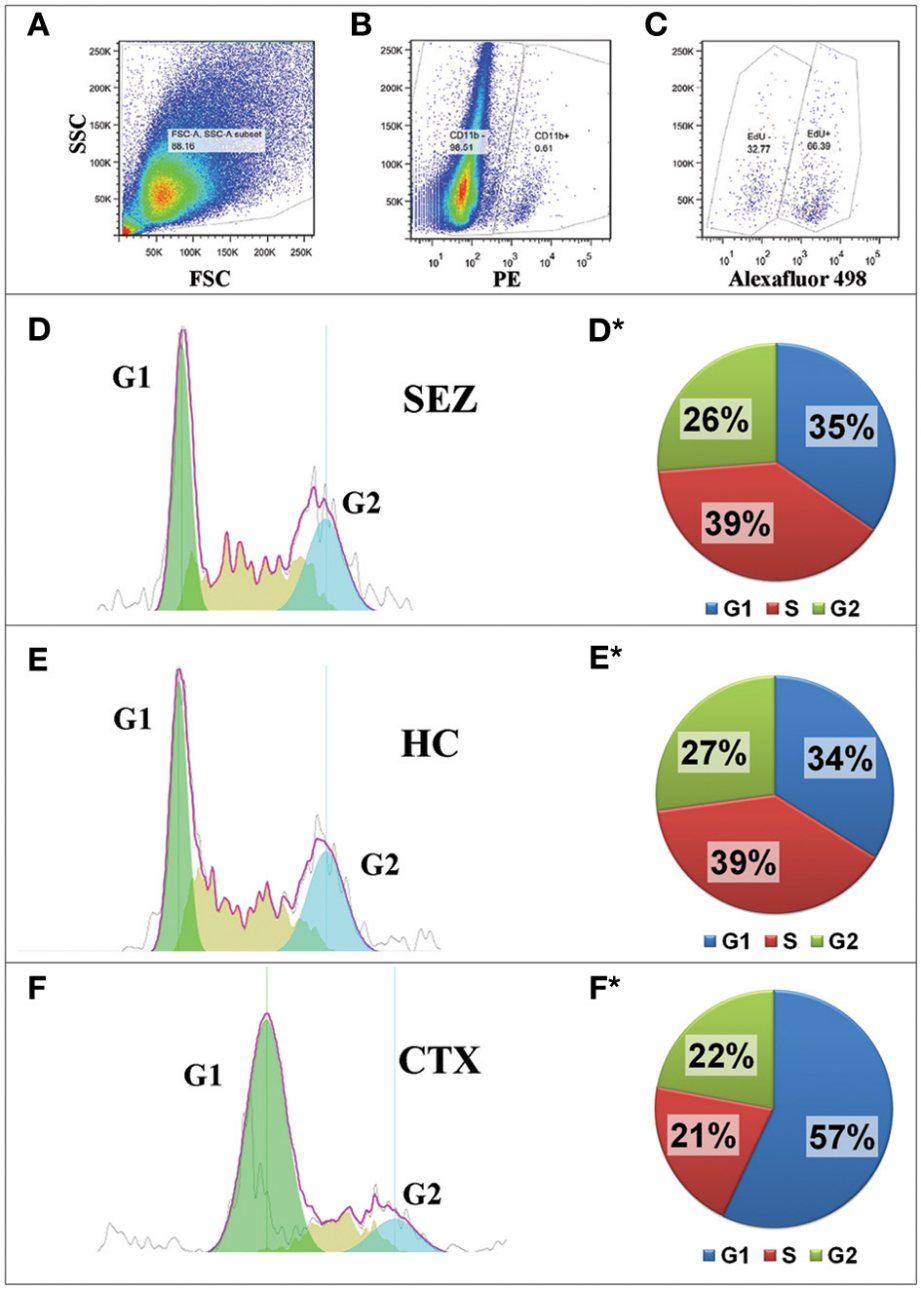
**Microglia from neurogenic regions are more proliferative than microglia from non-neurogenic regions**. Cultures (*n* = 1) from the neurogenic SEZ and HC, and from the non-neurogenic CTX were exposed to the thymidine analog, EdU, for 18 h. Cells were then fixed and labeled for EdU and the microglial marker, CD11b. In addition, total DNA was labeled with propidium iodide. Analysis by flow cytometry was as follows: debris was excluded from the Forward/Side scatter plot **(A)** before the CD11b+ fraction (microglia) was gated from the CD11b- fraction **(B)**; mitotic microglia were identified (EdU+) from the total microglia population **(C)**; and cell cycle was assessed using FlowJo software to analyze DNA content **(D–F)**. Cell cycle analysis reveals that the SEZ (D,D^*^) and the HC (E,E^*^) have remarkably similar numbers of mitotic microglia (roughly 65% in S/G2), whereas only 43% of the CTX microglia (F,F^*^) are mitotic. Differential proliferation between neurogenic and non-neurogenic regions is not seen with CD11b-negative cells (not shown), suggesting that the elevated proliferation rate is unique to microglia of the brain's neurogenic zone.

We have previously shown that both SEZ and HC primary cultures of both neonatal and adult dissociates are capable of large-scale microglia while the CTX is capable only of relatively modest microglia production that is limited to neonatal tissue (see Marshall et al., 2008 and Figure S1). We therefore hypothesized that there are differential cell cycle kinetics between microglia from neurogenic zones and microglia from non-neurogenic zones. To test this hypothesis we compared the cell cycle of microglia in cultures derived from the SEZ, HC, and CTX of neonatal mice. Confluent cultures of these regions were incubated with the thymidine analog EdU for 18 h and then processed for flow cytometric cell cycle analysis. The number of CTX microglia in G1 phase is substantially greater than the SEZ- or HC-derived microglia, while the number of SEZ and HC microglia in S phase is greater than CTX-derived microglia, indicating that more microglia within the SEZ and HC cultures are actively undergoing cell division (Figure 2). Interestingly, there are no statistically significant differences in the cell cycle profiles between the non-microglia cells (CD11b−/EdU+) among these three brain regions, suggesting that the regional cell cycle heterogeneity of microglia is not due to global differences inherent to the tissue source, but rather is specific only to the microglia population (data not shown). In addition, we assessed immunolabeling differences in mini chromosome maintenance protein 2 (Mcm2), which is a critical component of the pre-replication Mcm complex crucial for the onset of DNA replication and cellular division (Lei and Tye, 2001). Mcm2 is frequently used as a marker for cells capable of -or “primed” for- mitosis (Shetty et al., 2005). We assessed Mcm2 in microglia by co-immunolabeling with antibodies against CD11b and Mcm2. Sequential FACS analysis of the microglial populations at passages 3–5 reveals that, while the percentage microglia co- expressing Mcm2 decreases with each passage, the SEZ contains more Mcm2+ microglia than the passage-matched CTX (Figure S2). This finding, together with the cell cycle results, suggests that microglia from neurogenic regions possess a substantially greater potential for cellular division and population expansion than microglia from non-neurogenic regions.

### SEZ microglia expansion is suppressed by exposure to CTX

The massive expansion capacity of neurogenic zone microglia compared to non-neurogenic zone microglia may be due either to cell-intrinsic properties or to extrinsic cues within the environmental milieu of the NSC niche. We therefore asked whether the expansion capacity of neurogenic zone microglia is altered by exposure to a non-neurogenic environment (i.e., CTX-derived cultures). The experimental paradigm we used in this study is schematized in Figure S3. We generated mixed, heterotypic cultures by combining age-matched dissociated primary slurries from WT SEZ and GFP+ CTX at a 1:1 ratio (Figure S3 and Methods: Generation and Analysis of Heterospatial Adherent Cultures). Microglia expansion within these heterotypic cultures was compared to the expansion within un-mixed sister cultures of SEZ alone and CTX alone over the course of 4 passages. At Passage 1 the overall number of microglia in the heterotypic cultures was calculated in order to determine the relative starting contribution of microglia from the SEZ (GFP−) and CTX (GFP+). Twenty-four hours after plating, microglia composed 4% of the total adherent cell population, with roughly half of these being derived from the SEZ and half from the CTX (Figure S4).

**Figure 3:**
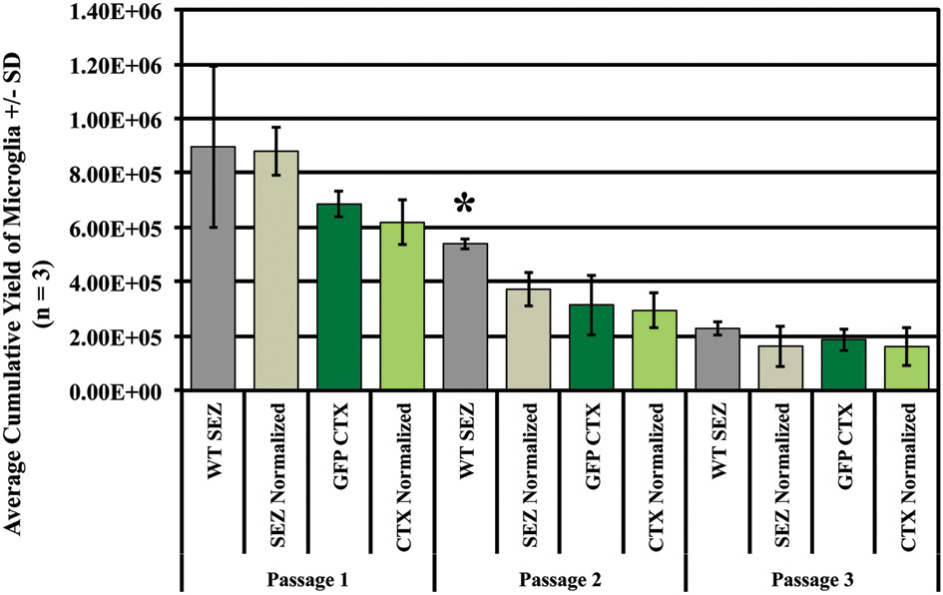
**Exposure to CTX cells *in vitro* suppresses SEZ microglia proliferation**. Primary cultures were established from 2 × 10^6^ SEZ cells (wild-type), 2 × 10^6^ CTX cells (GFP+), or from a mixture of 1 × 10^6^ SEZ + 1 × 10^6^ CTX cells (see schema in Figure S3). In order to determine if exposure to the SEZ “environment” increases proliferation of CTX microglia, isolation via mitotic shake-off was performed three times. The number and source of microglia were determined, and SEZ and CTX microglia isolated from mixed cultures were normalized by doubling to allow direct comparison to their unmixed controls. The total yield of microglia decreases with each isolation regardless of tissue source, but SEZ yield (dark gray bars) is consistently greater than CTX yield (dark green bars) over time. Exposure to SEZ cells does not significantly alter the yield of CTX microglia at any isolation (compare dark green bars to light green bars). Values for “GFP CTX” and “CTX normalized” are not statistically different; if exposure to SEZ environment enhances CTX microglia yield, then “CTX normalized” would be higher than “GFP CTX.” In contrast, exposure of SEZ microglia to the CTX environment seems to suppress SEZ yield (compare dark gray bars to light gray bars). In this case, “SEZ normalized” is lower than “WT SEZ” yield at passage 2 and 3, although only the difference at passage 2 reaches statistical significance. Yields were compared only within region and passage. One Way ANOVA. Asterisk indicates *p* < 0.05. *N* = 3 for all experiments.

In order to determine the effect of SEZ-CTX interactions on region-specific microglia yield independent of the total yield in mixed cultures, we assessed the percentage of GFP+ and GFP- microglia after isolation (shake-off) to determine the relative contributions from CTX and SEZ. We and others have previously shown that CD11b is a reliable marker for labeling all microglia in culture (Sedgwick et al., 1991; Marshall et al., 2008), and we used anti-CD11b antibody to label and identify the isolated microglia (Figure S5). These assays were performed in order to analyze the number and source of microglial populations over time as compared to the microglia present in the initial cultures. As expected, we found that unmixed SEZ cultures produce significantly more microglia than unmixed CTX cultures (Figure 3, compare WT SEZ to GFP CTX at isolations 1 and 2, *p* ≤ 0.05). In addition, at the first isolation SEZ microglia from mixed cultures (SEZ normalized) match the output of SEZ microglia from unmixed SEZ cultures (Figure 3, compare WT SEZ to SEZ normalized at isolation 1). However, with increased time *in vitro* mixed cultures produce significantly fewer SEZ microglia than the unmixed SEZ culture (Figure 3, compare WT SEZ to SEZ normalized at isolations 2 and 3, *p* ≤ 0.05), suggesting that exposure to the CTX environment reduces the expansion rate or capacity of SEZ microglia. In contrast, the expansion of CTX microglia in mixed cultures (CTX normalized) did not substantially differ from the CTX microglia output in unmixed cultures (GFP CTX) at any of the three passages.

### Donor SEZ microglia suppress host microglia expansion in homo- and hetero-spatial cultures

**Figure 4:**
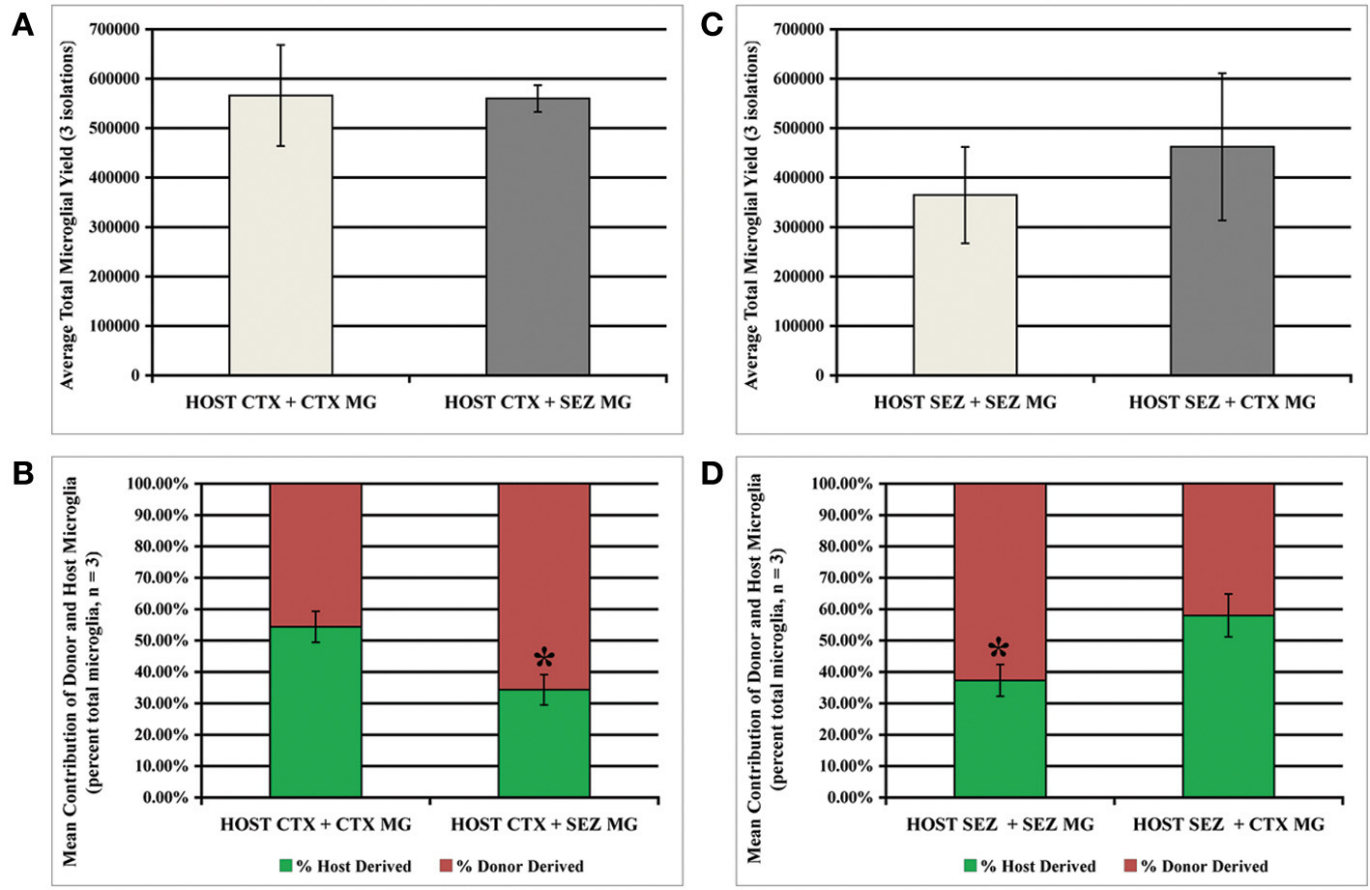
**Donor SEZ microglia suppress expansion of CTX and SEZ host microglia**. Established CTX or SEZ cultures (4 × 10^6^ GFP+ cells) were supplemented with 4 × 10^6^ wild-type “donor” microglia isolated from SEZ or CTX cultures (see schema in Figure S6) in order to assess the effect of large numbers of homo- and hetero-typic microglia on the expansion of the host microglia population. Beginning 1 week after supplementation three microglia isolations were performed at 3-day intervals. The tissue source of donor microglia did not significantly affect total microglia yield from either CTX host **(A)** or SEZ host **(C)**. The relative contribution of microglia from donor and host was not affected by supplementation with CTX microglia, with statistically equal numbers of isolated wild-type and GFP+ microglia (**B**, left bar & **D**, right bar). However, supplementation with SEZ microglia reduced the donor microglia output from both CTX (asterisk in **B**) and SEZ (asterisk in **D**). *p* < 0.05 for both **(B)** and **(D)**, One Way ANOVA with Bonferroni's Multiple Comparison Test, (*n* = 3).

The previous experiment with directly mixed primary dissociates indicates that the normal high rate of SEZ microglia expansion is attenuated by exposure to the CTX environment, while expansion of CTX microglia is unaffected by exposure to the SEZ environment. We hypothesized that the relative ratio of SEZ to CTX microglia may be an important variable for altering the proliferative behavior of CTX microglia, and therefore asked if the introduction of purified, expanded microglia to CTX cultures can improve CTX microglia expansion. By exploiting the *in vitro* expansion capacity of SEZ microglia this approach allows us to increase the ratio of donor-to-host microglia far beyond what can be achieved by mixing primary dissociates from different regions. As schematized in Figure S6 (also see Methods: Surface Supplementation of Established Adherent Cultures with Homo- and Hetero-typic Isolated Microglia) we seeded 1 × 10^6^ passage 1 WT SEZ “donor” microglia onto confluent “host” GFP+ cultures derived from either the SEZ (homo-spatial) or CTX (hetero-spatial). Likewise, 1 × 10^6^ passage-1 WT CTX donor microglia cells were seeded onto confluent GFP+ CTX (homo-spatial) and SEZ (hetero-spatial) host cultures. All cultures were maintained in standard NGM for 1 week, then transferred to MPM for 3 days. Microglia were then isolated, quantified and analyzed for the relative proportion of GFP+ microglia, as described above. We found that CTX cultures supplemented with either homo- or hetero-spatial microglia produce nearly identical total numbers of microglia (Figure 4A). However, within this total microglia population the proportion of donor-to-host derived microglia is significantly different. While homo-spatial CTX cultures produce nearly equivalent numbers of donor and host microglia, within heterotypic cultures 66% of the microglia are SEZ donor-derived (Figure 4B; *p* < 0.05). Likewise, SEZ cultures supplemented with either homo- or hetero-spatial microglia produce similar total numbers of microglia (Figure 4C). But here again, a significant difference is observed when the donor source is considered. Within heterotypic SEZ cultures the microglia population consists of nearly equal proportions of donor and host microglia. In contrast, 63% of the microglia in the homotypic cultures are derived from the donor SEZ microglia (Figure 4D; *p* < 0.05). To summarize, the presence of SEZ microglia does not enhance the capacity for CTX microglial expansion, indicating that the limited expansive potential of CTX-derived microglia cannot be enhanced by the proximity of SEZ-derived microglia or the factors they may secrete. In fact, SEZ-derived donor microglia inhibit the expansion of CTX and SEZ endogenous microglia populations.

### Repeated SEZ microglia supplementation promotes neuroblast survival

Primary SEZ dissociates from heterogeneous adherent cell mixtures *in vitro* consist predominantly of astrocytes and microglia, but also contain a small population of neuroblasts (small β-III tubulin+ cells with rounded somas and short processes) that disappear upon repeated passaging (Scheffler et al., 2005). This loss of neurons is concomitant with declining numbers of microglia (unpublished observation), suggesting a role for microglia in maintaining neuronal survival in culture. Since these neuroblasts are unique to the SEZ we hypothesized that supplementing long-term, multi-passage SEZ cultures with microglia isolated from early-passage SEZ cultures would ameliorate the passage-related loss of neuroblasts, and that this protection would not be conferred by supplementation with early-passage CTX microglia. The experimental paradigm is schematized in Figure S7 (also see Methods: Microglia Supplementation of SEZ Cultures). SEZ cultures were generated from WT neonatal mice and grown to confluence. At the first passage, microglia isolated from either the SEZ or CTX of age-matched GFP mice were introduced to the suspended SEZ culture at 10% of the total cells prior to plating. This percentage was chosen since we have determined that the average percentage of microglia present in primary SEZ dissociates is ~7–10% (data not shown). Cultures were supplemented with microglia and neurons were quantified at each of the first three passages. Additionally, test cultures were assessed at each passage for overall microglia yield 24 h after plating.

**Figure 5:**
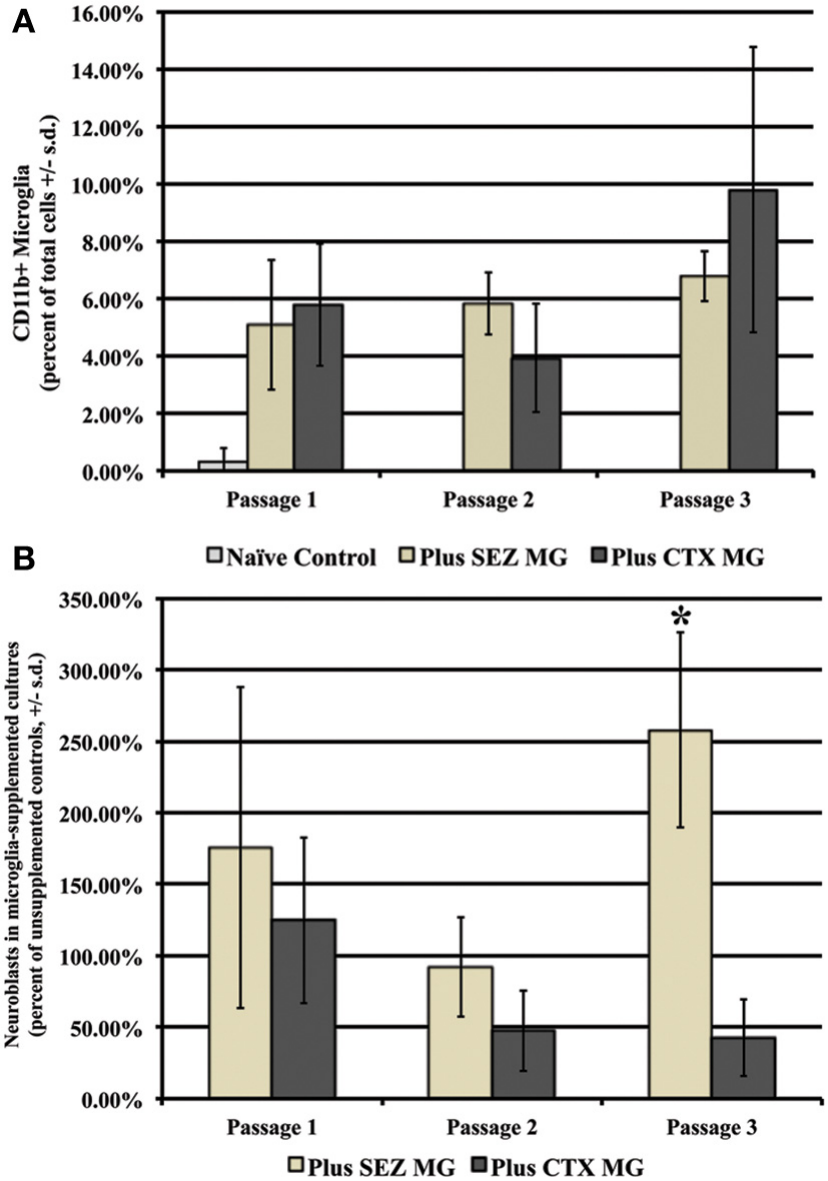
**Supplementation with SEZ but not CTX microglia increases neuroblast yield within SEZ cultures**. First passage microglia isolated from either SEZ or CTX were added to established SEZ cultures at passages 1, 2, & 3. Both groups contained significantly more total microglia than unsupplemented control cultures at each passage, but contained similar levels of microglia as compared to each other **(A)**. While no significant difference in the number of neurons was observed in supplemented cultures at early passages, by passage 3 cultures supplemented with SEZ derived microglia contained significantly more neuroblasts than both unsupplemented cultures and cultures supplemented with CTX-derived microglia **(B)** (One Way ANOVA, ^*^p ≤ 0.05, *n* = 3).

As expected, supplementation with either SEZ-derived or CTX-derived microglia results in significantly more total microglia at each of three passages, although no significant differences are observed between supplemented groups (Figure 5A). Repeated introduction of expanded SEZ-derived microglia into sequentially passaged SEZ cultures preserves neuroblast levels; by passage 3 there are significantly more neuroblasts as compared to both unsupplemented controls and to CTX- supplemented SEZ cultures, suggesting that SEZ microglia are uniquely capable of providing neurotrophic support to immature neurons (Figure 5B).

### Repeated SEZ microglia supplementation preserves inducible neurogenesis

**Figure 6:**
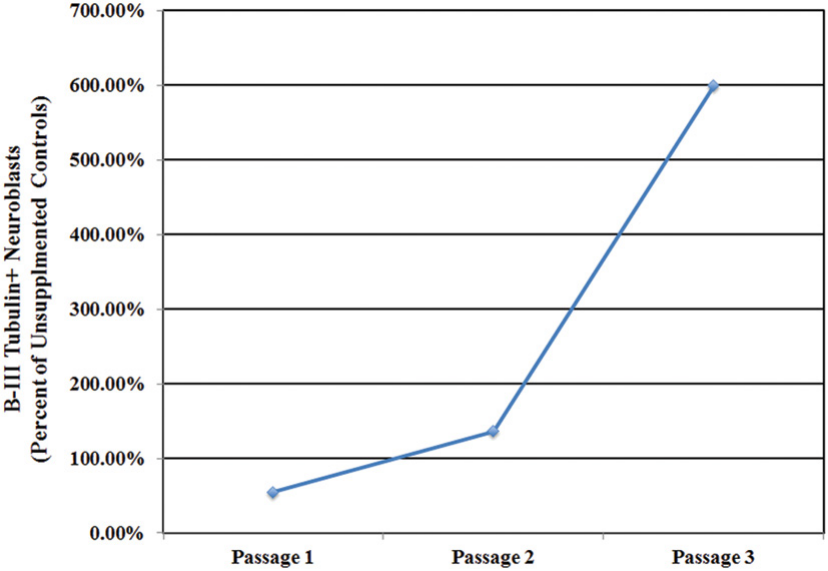
**Microglia supplementation preserves inducible neurogenesis**. The repeated introduction of GFP+ SEZ-derived microglia into WT SEZ cultures prevents the passage-related decline in IN evident at passage 3 in control cultures, with neuroblast levels noticeably higher in supplemented cultures (*n* = 1 for control and treated cultures at each of three passages).

**Figure 7:**
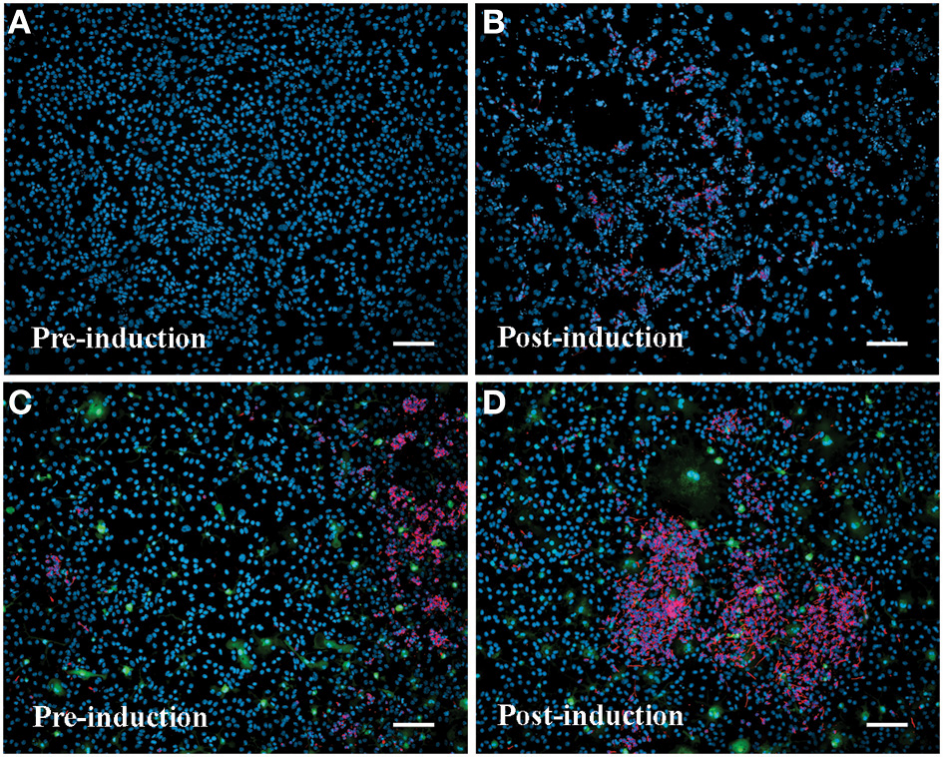
**Supplementation with SEZ microglia enhances inducible neurogenesis in SEZ cultures**. Passage 3 control SEZ cultures in standard medium contain few or no neurons **(A)**. Following induction (withdrawal of serum and mitogens) control SEZ cultures show a small increase in neurogenesis **(B)**. In contrast, passage 3 SEZ cultures supplemented with SEZ microglia contain numerous neurons prior to induction **(C)**, and this level is dramatically increased after the induction protocol **(D)**. Scale bars = 50 μm; blue = DAPI, red = β-III tubulin+ neurons, green = GFP+ microglia.

Inducible neurogenesis (IN) from adherent SEZ cultures is a unique phenomenon of neurogenic astrocytes, whereby the withdrawal of mitogens and serum results in the de novo production of neuroblasts (Scheffler et al., 2005, and Figure S8). The capacity for IN may be linked to the presence of microglia, since the robustness of IN decreases concomitantly with diminishing microglia numbers over multiple passages and is eventually lost in high passage cultures that invariably contain fewer microglia. Additionally, there is evidence that IN can be restored by conditioned medium derived from microglia-rich, early-passage SEZ cultures (Walton et al., 2006). Here we asked if repeated supplementation of SEZ cultures with homotypic SEZ microglia can reduce or prevent the normal loss of IN that occurs during sequential passaging of SEZ cultures. The experimental paradigm is schematized in Figure S9 (also see Methods: Supplementation of Neurogenic Cultures with Homotypic Microglia). Host cultures were derived from WT SEZ, as described above. At each passage purified microglia, isolated from age- and passage-matched GFP+ SEZ cultures, were again added at 10% of the total cell population. At each passage supplemented cultures were assessed for IN potential. Unsupplemented, passage-matched sister cultures served as controls. Sequential supplementation of SEZ cultures with isolated SEZ microglia leads to progressively increasing levels of IN as compared to unsupplemented sister cultures. Graphical representation of neuroblast production (Figure 6) shows that IN at passage 1 is lower than, but not statistically different from, the unsupplemented control. By passage 2 the supplemented group shows slightly higher IN than control, but again this difference is not statistically different. By passage 3, however, IN within the supplemented group is substantially and statistically higher than control. Photodocumentation of passage 3 IN clearly shows the robust difference in neuroblast production between unsupplemented and supplemented cultures (Figure 7; compare **A,B** to **C,D**). Interestingly, pre-induction supplemented cultures contain substantially more neuroblasts than pre- induction controls (compare Figure 7C to Figure 7A), presumably due to the increased neurotrophic effect of SEZ microglia as described in the previous experiment.

**Figure 8:**
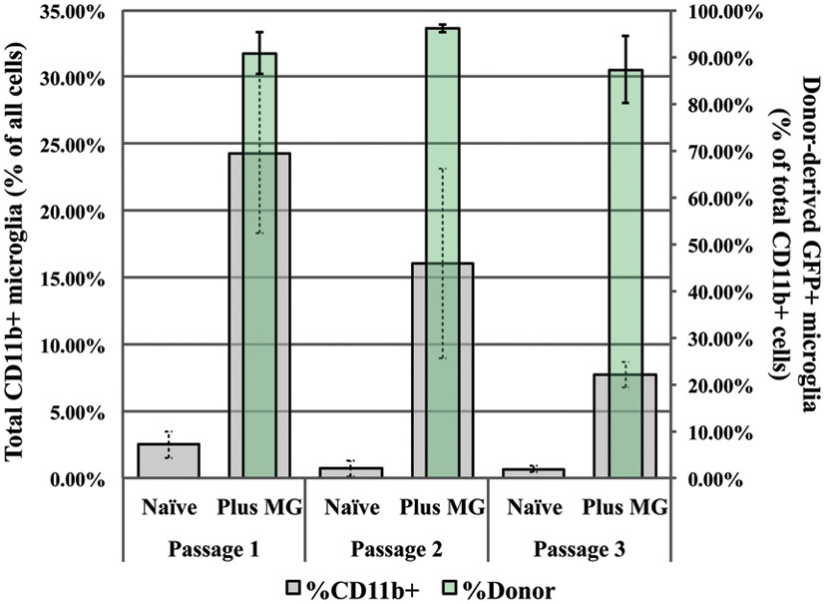
**Analysis of microglia populations in supplemented and naïve control cultures**. Naïve cultures contain no more than 3% microglia, while nearly 25% of the cells in passage 1 supplemented cultures are microglia (gray bars, left y-axis). By passage 3, at the same time in which inducible neurogenesis is significantly restored, supplemented cultures are comprised of only 7% microglia. Yet at all passages, donor microglia in supplemented cultures comprised ~90% of all microglia present (green bars, right y-axis, *n* = 3).

We also assessed the relative numbers of microglia present in both supplemented and unsupplemented controls at each passage. At Passage 1, microglia represent no more than 3% of all cells in the unsupplemented culture, but nearly 25% of all cells in the supplemented cultures (Figure 8, gray bars and left-side ordinate). Of this 25%, roughly 90% of are donor-derived (green bars and right-side ordinate), indicating substantial proliferation by the donor microglia. At each subsequent passage the total microglia population is diminished in both supplemented and unsupplemented cultures, while the relative contribution by the donor microglia remains constant (~90%). At passage 3 the total number of microglia present in the supplemented cultures is 8% compared to less than 1% in naïve cultures (Figure 8), correlating with the restored neurogenic potential seen in Figure 7. This finding supports the hypothesis that SEZ-derived microglia can preserve IN, and further suggests that the ratio of microglia to total cells may be important for determining the neurogenic potential of SEZ cultures.

## Discussion

Microglia have traditionally been thought of as functionally homogenous across brain regions. That is, while there may be some functional differences between infiltrating perivascular microglia and endogenous parenchymal microglia (Hickey and Kimura, 1988), the functions of microglia do not change substantially as one considers the different anatomical structures of the neuraxis. However, recent reports have begun to challenge this view, particularly with respect to unique functions of microglia in the neurogenic zones of the mammalian brain. Goings et al. (2006) showed that SEZ microglia and non-neurogenic zone microglia *in vivo* differ with respect to basal levels of proliferation as well as constitutive and injury-induced markers of activation. Additionally, our group has reported that SEZ microglia are capable of up to 20-fold greater *in vitro* expansion than non-neurogenic zone microglia (Marshall et al., 2008). In the present study we further examined the regionally specified functional variation between microglia from neurogenic and non-neurogenic regions of the mouse brain. Specifically, we assessed differential cell cycle kinetics and support of neurogenesis by these two broad categories of microglia.

### Microglia display regional variation in cell cycle kinetics

Previous studies have suggested that microglia within the SEZ are more proliferative than microglia in other brain regions based upon the uptake of BrdU *in vivo* (Goings et al., 2006) as well as their capacity for large scale expansion *in vitro* (Marshall et al., 2008). However, the cell cycle kinetics of regionally discrete populations of microglia has not been reported. Our flow cytometry results are consistent with these antecedent studies in showing that a substantially higher percentage of microglia from the neurogenic SEZ and hippocampus are actively dividing compared to CTX microglia. We additionally assessed the protein density of the Mcm2 in SEZ vs. CTX microglia. The minichromosome maintenance protein family regulates the initiation of chromosomal replication in eukaryotic cells, and they can be expected to be expressed in cells that are competent to divide (Todorov et al., 1991; Kelly and Brown, 2000). As expected from the flow cytometric data, SEZ microglia express higher levels of Mcm2 than CTX microglia over multiple passages, confirming fundamental, spatially restricted differences in proliferative activity between neurogenic region and non-neurogenic region microglia. Because of morphological and immunophenotypic overlap, our studies do not distinguish between microglia and circulating macrophages. However, our results are consistent with antecedent *in vivo* work with parenchymal microglia. In addition, regional differences functions still apply, and still indicate functional differences between neurogenic and non-neurogenic brain regions. Further, increased neurogenic region yield cannot be accounted for by the potential presence of greater numbers of macrophages in neurogenic regions, since all of our quantification was normalized when we calculated fold expansion.

### Proliferative characteristics of microglia are influenced by environmental cues

The finding that neurogenic zone and non-neurogenic zone microglia differ with respect to proliferative activity raises the question of whether this difference is intrinsic to the separate populations of microglia, or whether the difference is due to the specific microenvironment that the microglia are exposed to. Specifically, is there something about the neurogenic niche of the brain that promotes microglial proliferation, or maintains microglia in a competent state to divide? The idea that neurogenic regions of the brain might be uniquely supportive of proliferation was suggested by experiments showing the existence of a unique vascular niche within the hippocampus that seems to be the proliferative focus of neurons, glia, and endothelial cells (Palmer et al., 2000). Conversely, it may be that the microenvironment of non-neurogenic regions suppresses or fails to support the intrinsic proliferative capacity of microglia residing there. Thus, we performed two types of co-culture experiments to determine whether exposure to the SEZ culture microenvironment improves the proliferation dynamics of CTX microglia and—vice versa—whether exposure of SEZ microglia to the CTX environment reduces proliferation dynamics. Obviously, the *in vitro* microenvironment is not an exact replica of the complex *in vivo* environment; nevertheless, we hypothesized that these two approaches would provide a reasonable simulacrum sufficient to reveal gross effects on proliferation. Indeed, both studies show clear and consistent environmental effects, arguing for the validity of this approach. Specifically, in both experiments SEZ microglia proliferation is suppressed by exposure to the CTX environment, either by direct mixing of primary dissociates (Figure 3) or by supplementation of SEZ cultures with purified CTX microglia (Figure 4). Conversely, in neither instance is the proliferation of CTX microglia altered by exposure to the SEZ environment. These data can be interpreted to indicate that SEZ and CTX microglia do possess intrinsically different programs of proliferation given a permissive environment, and that some aspect of the CTX environment is non-permissive for the SEZ program. Alternatively, it may be that differential proliferation between SEZ and CTX microglia results from regional environmental cues, and that some aspect of our culture paradigm allowed the CTX environment to alter SEZ microglia but not for the SEZ environment to alter CTX microglia. This may be due to a greater sensitivity of SEZ microglia to CTX cues, or perhaps to the necessity for a longer exposure time for CTX microglia to respond to SEZ cues.

From these data we conclude that our co-culture paradigms preserve at least some relevant *in vivo* environmental cues, and that these cues are functionally sufficient to alter microglia proliferation dynamics. Surprisingly, the microglia supplementation approach (Figure 4) showed that the proliferation rate of SEZ “host” microglia are suppressed by addition of both CTX and SEZ “donor” microglia. This results seems counterintuitive both because it is not expected that SEZ should inhibit SEZ, and because CTX “host” microglia are not suppressed by the addition of “donor” SEZ microglia. It may be, however, that SEZ microglia are uniquely sensitive to the relative ratio of microglia within a region. This interpretation is supported by our data showing that *in vitro* microglia-mediated support of neuroblasts is maximal within a narrow proportion of microglia to total cells (discussed below). A similar negative regulation may operate in the supplementation study, whereby “host” SEZ microglia reduce proliferation in an attempt to maintain a particular ratio within the culture environment.

### SEZ microglia but not CTX microglia support *in vitro* neurogenesis

There is compelling evidence that microglia and associated immune factors influence neurogenesis both *in vivo* and *in vitro*. Beck et al. (2005) reported that hippocampal neurogenesis in transgenic mice lacking the cytokine IL-2 was significantly increased in comparison to littermate controls (although this effect was seen only in males). The authors proposed that this increase resulted from dysregulation of the mouse's neuroimmunological status acting on neuronal progenitor cells of the dentate gyrus. Another study, using an environmental enrichment paradigm known to augment adult hippocampal neurogenesis, showed that microglia activation was associated with increased neuronal production (Ziv et al., 2006). These authors also reported that immune-deficient SCID mice display impaired levels of neurogenesis under normal conditions, and that these levels are not responsive to environmental enrichment. Using an adrenalectomy model, Battista et al. (2006) were able to correlate increased hippocampal neurogenesis with the number of activated microglia in the dentate gyrus. Interestingly, they also found that this increased neurogenesis was likely due, at least in part, to the effects of TGFβ —an anti-inflammatory cytokine secreted by microglia. It has been reported that neurogenesis can either be blocked or enhanced by the action of microglia depending upon how they are activated (Butovsky et al., 2006). Although initial studies in rodents suggested that neuroinflammation (microglial activation) was detrimental to neurogenesis (Ekdahl et al., 2003; Kempermann and Neumann, 2003), more recently the tide of opinion has shifted and the prevailing notion now appears to be that the role of neuroinflammation is “much more complex” (Ekdahl et al., 2009). A number of publications suggest a beneficial role for microglia in the regulation of the migration, proliferation, and differentiation of neural stem/progenitor cells (Battista et al., 2006; Butovsky et al., 2006; Walton et al., 2006). More recently, Vukovic et al. (2012) have reported that exercise induced neural precursor activation in the hippocampus is mediated through the CX3CL1-CX3CR1 pathway. This signaling axis is reported to be critical for modifying the phenotype of microglia residing in the hippocampus toward a phenotype that increases neural progenitor cell activity. As CXCL1 is detectable on neurons of the dentate gyrus and exercise elevates protein density, the authors speculate that this may be the means by which regions of neurogenesis can direct neighboring microglia (which express CX3CR1 on their cell surface) to support neurogenesis, and may help explain why microglia from different regions of the brain perform different roles. One reason for the initially negative view on the neuroinflammation/neurogenesis nexus is likely to be found in the use of intraparenchymal or systemic administration of bacterial lipopolysaccharide (LPS) to induce widespread and non-specific inflammation, ignoring potentially direct toxic effects of LPS on neural cells (Monje et al., 2003; Lee et al., 2005). The sudden administration of LPS to experimental animals induces a condition similar to acute septic shock and it therefore does not mimic physiologically relevant neuroinflammation (Cohen, 2002).

In our present study we examined microglia support for neuroblast production in two *in vitro* models. Because both neuroblasts and microglia disappear from primary SEZ cultures upon repeated passaging, we first tested the effect of repeated microglia supplementation on the survival of SEZ neuroblast. We hypothesized that by adding microglia at each passage (at 7–10% of the total cell population as is seen in primary dissociations) we would enable neuroblasts to persist in culture longer and at greater numbers. Our data, represented in Figure 5, show that supplementation with SEZ microglia does result in increased numbers of neuroblasts over three sequential passages. In contrast, supplementation with equal numbers of CTX microglia results in fewer neuroblasts as compared to control, though these differences are not statistically significant.

We next performed a second assay designed to assess the effect of repeated SEZ microglia supplementation on inducible neurogenesis from SEZ cultures. Here, too, SEZ microglia supplementation leads gradually to an increase in inducible neurogenesis such that, by the third passage, there is a 6-fold increase in induced neuroblast formation. It is important to note that purity analysis shows that we are not introducing additional neuroblasts when we supplement with isolated microglia (Marshall et al., 2008 and Figure S3). Interestingly, this study also revealed that the relative proportion of microglia within the culture is important for determining functional effects, with inducible neurogenesis maximized when microglia represent between 5 and 10% of the total cell population. This ratio—remarkably similar to that seen in primary dissociates—may represent an optimal range for microglial regulation of neurogenesis.

Our analyses cannot discern the role that microgla proliferation, *per se*, plays in neuroblast support. That is, is proliferation itself required for this support, or is it high proliferation rate correlated with other aspects of microglia biology that support neuroblasts? Because we are studying co-cultures, we are currently unable to selectively block microglial proliferation without also perturbing neuroblast precursors contained within the culture.

## Conclusion

From our results we conclude that microglia from neurogenic regions have a vastly greater proliferative cell cycle profile than microglia from non-neurogenic regions; furthermore, the higher proliferative capacity of neurogenic microglia can be modulated by the surrounding cellular environment, suggesting that it results at least partially from cell-extrinsic factors. We also conclude that neurogenic zone microglia are uniquely capable of supporting neuroblasts *in vitro*, although this support is not linear but rather depends upon the relative density of microglia within the cellular milieu.

### Conflict of interest statement

The authors declare that the research was conducted in the absence of any commercial or financial relationships that could be construed as a potential conflict of interest.
