# Supplementary material for: Microglia from neurogenic and non-neurogenic regions display differential proliferative potential and neuroblast support
Source: Front Cell Neurosci. 2014 Jul 16;8:180. doi: 10.3389/fncel.2014.00180 (PMC4100441; doi:10.3389/fncel.2014.00180)
Supplement: Supplementary file 1 [file Presentation1.PDF]

Microglia from neurogenic and non-neurogenic regions display differential proliferative potential and neuroblast support

Gregory P. Marshall II[1], Loic Deleyrolle[2], Brent A. Reynolds[2], Dennis A. Steindler[2] and Eric D. Laywell[3]

Departments of Anatomy and Cell Biology[1] and Neurosurgery[2], College of Medicine, University of Florida, Gainesville, Florida USA

and

Department of Biomedical Sciences[3], College of Medicine, Florida State University, Tallahassee, Florida USA

Running title: Microglia Display Regional Functional Heterogeneity

Address correspondence to:

Eric D. Laywell, PhD
Department of Biomedical Sciences
College of Medicine
Florida State University
1115 West Call Street
Tallahassee, Florida 32306
email: eric.laywell@med.fsu.edu

Key Words: microglia, inducible neurogenesis, proliferation, functional heterogeneity, subependymal zone

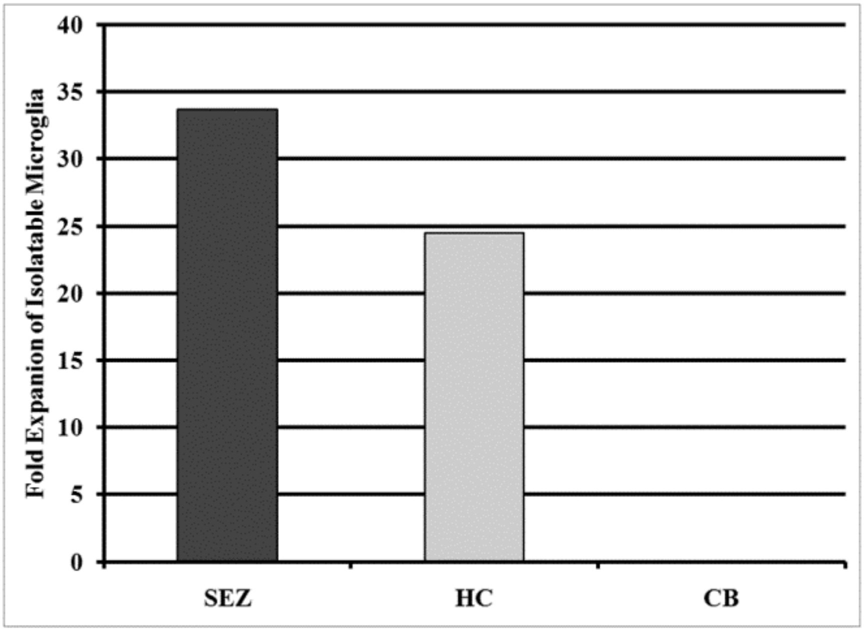

Supplemental Figure 1: Fold expansion of microglia from cultures derived from post-natal day 15 mice. The post-neurogenic CB is incapable of producing isolatable microglia, while the persistently neurogenic SEZ and HC generate expandable cultures capable of producing large number of isolatable microglia.

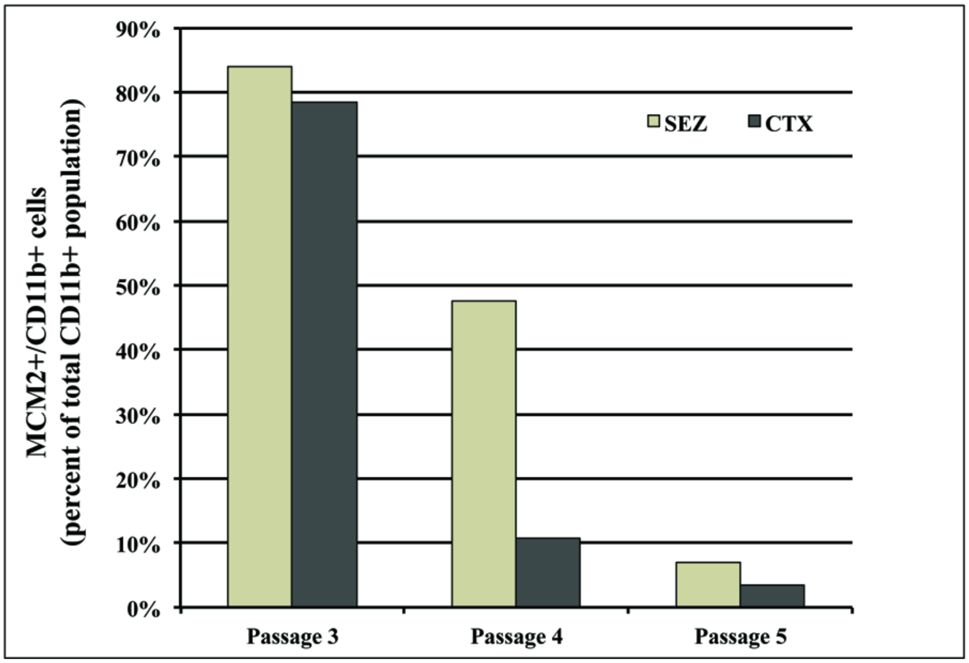

Supplemental Figure 2: SEZ cultures contain more MCM+ microglia than CTX cultures. Sequential flow cytometric analysis of passage 3-5 SEZ and CTX cultures was performed to assess the percentage of microglia (CD11b+ cells) from each region expressing the MCM2 transcription factor, indicating competence to divide. The ratio of MCM2+ microglia to total microglia decreases with passaging, but SEZ cultures consistently contain a higher percentage of MCM2+ microglia than CTX cultures.

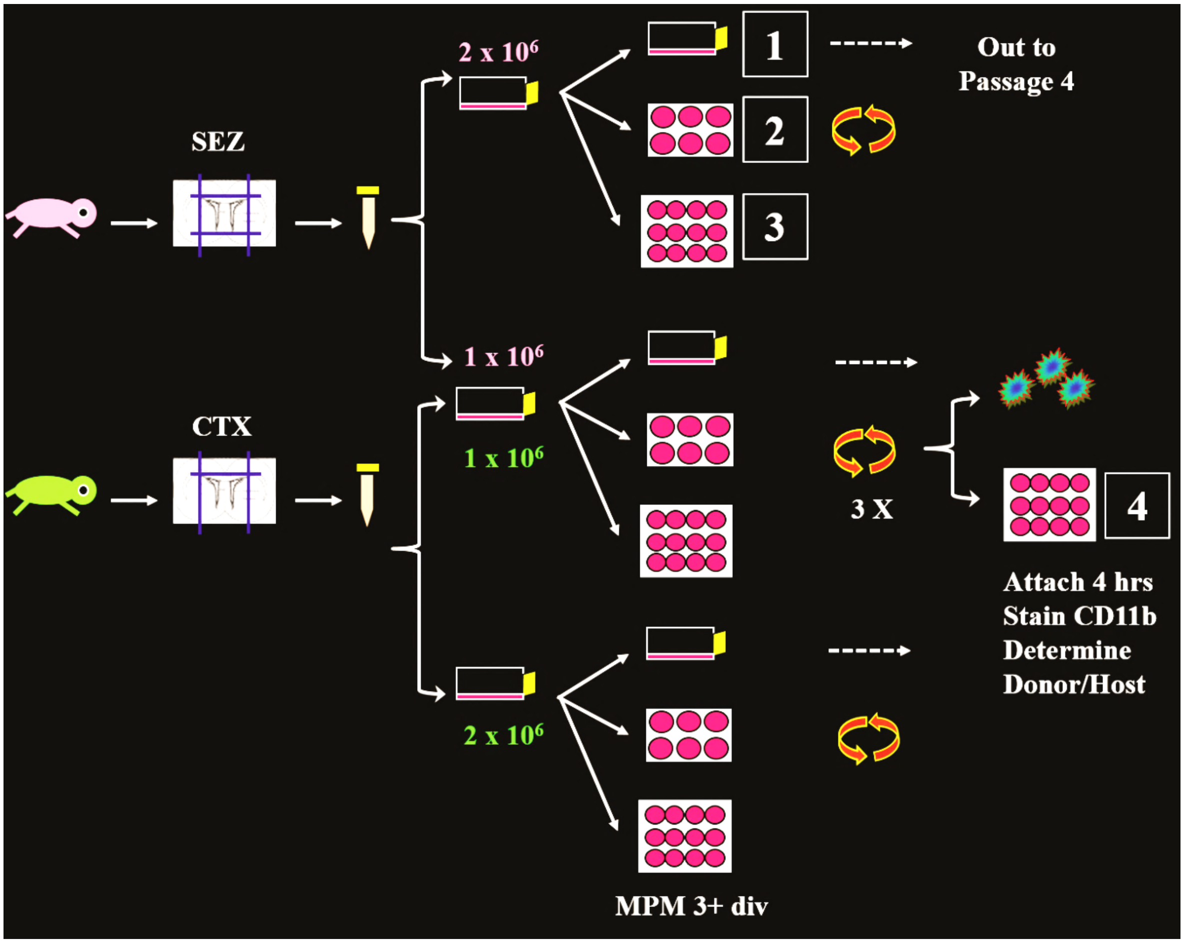

Supplemental Figure 3: Schema for generation and analysis of microglia expansion in mixed cultures. Age-matched neonatal WT and GFP+ mice were sacrificed and their brains dissected as described above. WT SEZ and GFP+ CTX were removed and processed independently into dissociated slurries as described above. Cells werequantified using a hemacytometer and Trypan blue exclusion. 2x10^6 cells were used to generate independent WT SEZ or GFP+ CTX cultures. In addition, mixed SEZ/CTX cultures were generated by combining 1x10^6 cells of the primary dissociate from each region. Upon confluence, cells were passaged to generate three separate groups of daughter cultures: [1] a maintenance culture for continued passaging (1x10^6 onto T-25 flasks); [2] cultures for the assessment of microglial isolation and expansion (25,000 cells/cm2 in 10 cm2 dishes); and [3] a culture for differentiation and phenotype assessment (50,000 cells plated onto P-Orn coated coverslips, 3.8cm2). Culture [3] was fixed 24hrs after plating, and immunolabeled with anti-CD11b to label and quantify microglia as described below. The total number of cells in each field was determined by nuclear counter stain (DAPI), and the number of WT and GFP+ microglia was determined. Thus, we assessed the total number of microglia present at each passage, as well as the overall contribution to the microglial population from both the SEZ (GFP-) and CTX (GFP+). Culture [2] was maintained until confluence at which time the medium was replaced with MPM and microglia were isolated by shake-off as described above. Three serial isolations at three day intervals were performed from each culture, with fresh MPM applied to the cultures following each isolation procedure. Isolated microglia were counted with a hemacytometer, plated onto P-Orn coated coverglass for 24hrs, and stained for CD11b to assess the relative SEZ (GFP-) and CTX (GFP+) contributions to the total microglia population [4]. Culture [1] was maintained until confluence at which time the culture was passaged to repeat these analyses.

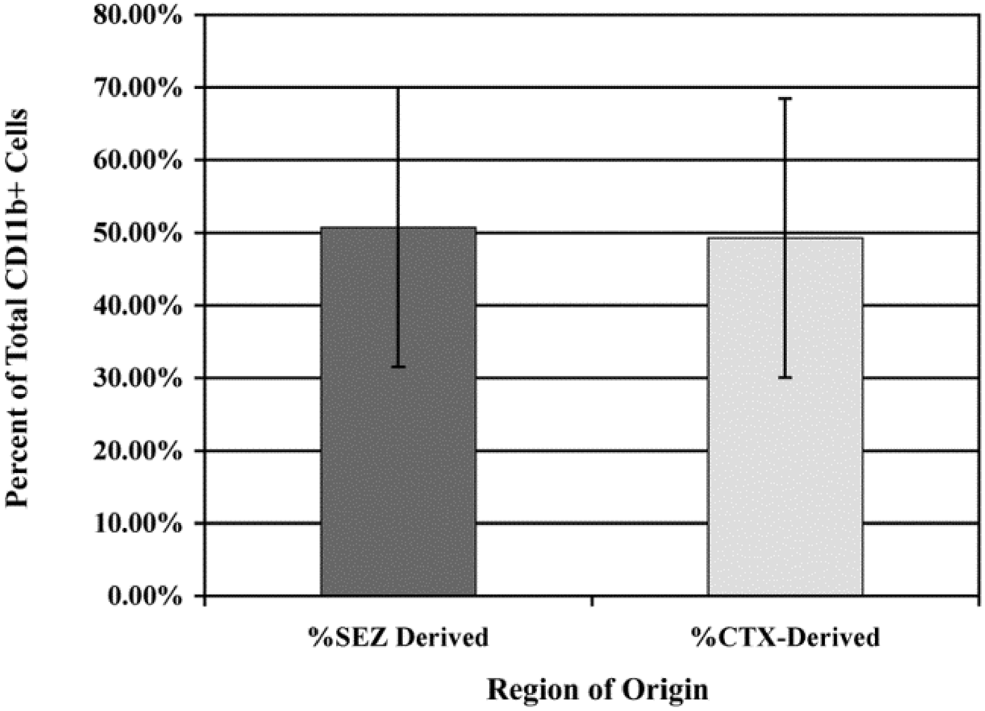

Supplemental Figure 4: Mixed Cultures Initially Contain Equal Numbers of SEZ and CTX Microglia. Passage 1 SEZ /CTX mixed cultures were assessed 24 hours after plating for expression of the microglial specific marker CD11b. CD11b+ microglia comprised 4% of the total adherent population. Based upon the expression of GFP, the source of the CD11b+ microglia could be determined as either SEZ (GFP-), or CTX (GFP+). No statistically significant difference in GFP+ versus GFP- microglia is evident (n = 5).

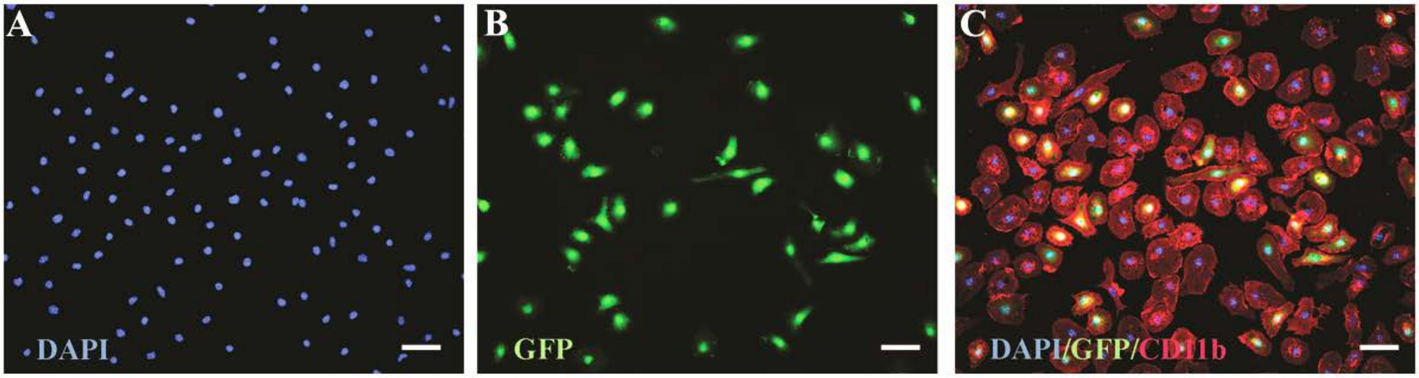

Supplemental Figure 5: Distinguishing microglia source by GFP expression. Microglia isolated mixtures of CTX (GFP+) and SEZ (GFP-) dissociates were allowed to attach to coverslips for 6 hours before being immunolabeled for CD11b. All cells were positive for the microglial marker CD11b (red, C), with only a subpopulation expressing GFP (green in B, yellow in C), allowing for the calculation of relative microglia contribution from the two brain regions. Scale bar in all images set at 50µm. (A) Nuclei stained with DAPI (Blue).

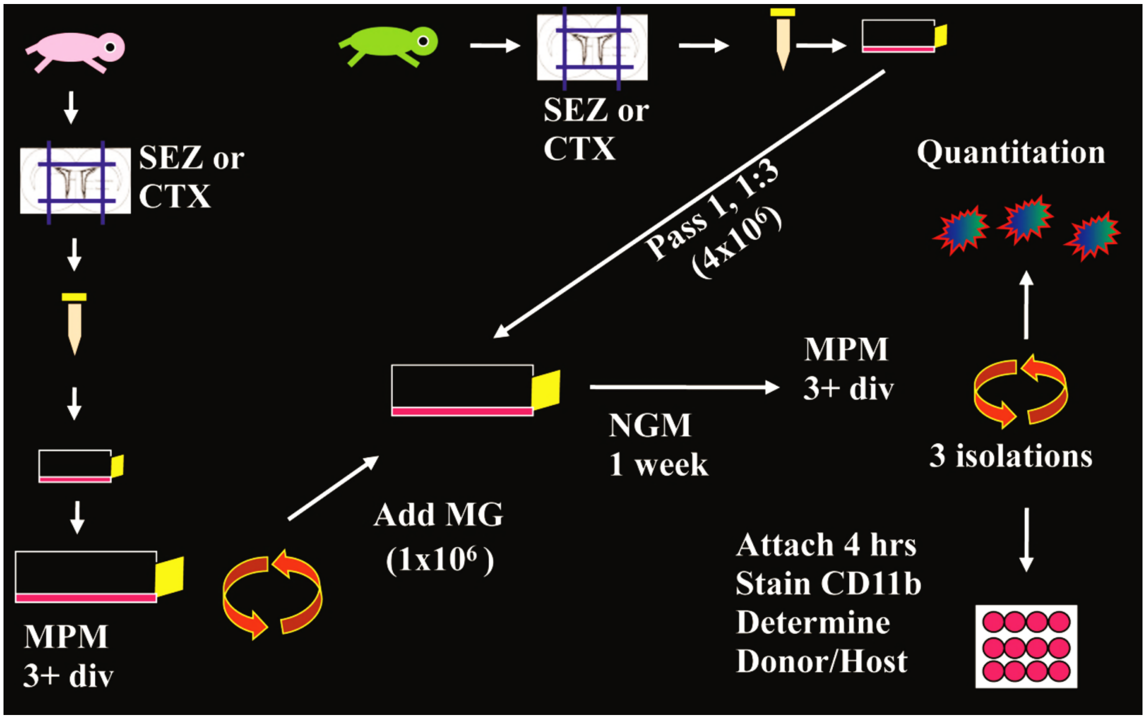

Supplemental Figure 6: Schema for supplementing adherent cultures with homo- and hetero-spatial "donor" microglia. Adherent cultures derived from the SEZ and CTX of GFP+ and WT neonatal mice were prepared as described above. Microglia expansion and isolation was induced in confluent Passage 1 WT SEZ and CTX cultures as described above. Aliquots from both cultures were processed for the purpose of quantification and immunophenotyping (see above). Additionally, $1x10^6$ purified microglia were seeded onto the surface of existing confluent Passage 1 cultures established from the SEZ and CTX of neonatal GFP+ mice in the following manner: 1) WT SEZ microglia onto both GFP+ SEZ and GFP+ CTX cultures (homo- and hetero-typic cultures, respectively); and 2) WT CTX microglia onto both GFP+ SEZ and GFP+ CTX cultures. Cultures were maintained in NGM for 1 week, then the medium was replaced with MPM for 3 days and the microglia were isolated by shake-off. At each isolation the microglia were quantified and plated onto P-Orn coated coverslips for 24 hours, then fixed and stained for the microglia marker CD11b as described below. CD11b+ microglia were additionally assessed for GFP expression to determine the host versus donor origin. Control experiments were performed in which the donor and host cultures sources were reversed (i.e., donor microglia were derived from GFP+ SEZ and were plated onto WT SEZ adherent cultures), and no effect of the GFP transgene was observed.

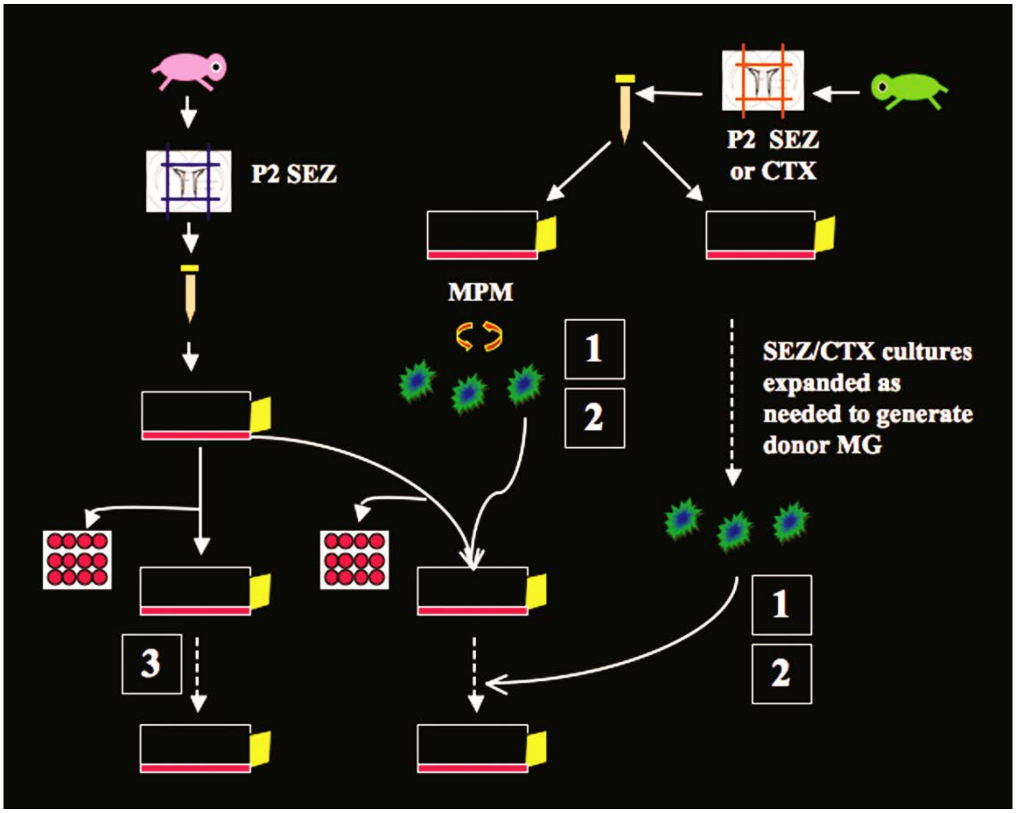

Supplemental Figure 7: Schema for repeated supplementation of adherent cultures with homo- and hetero-spatial microglia. SEZ and CTX cultures were generated as above from neonatal GFP+ mice for the purpose of microglia expansion and isolation. The cultures were expanded so that one sister culture was used for subsequent expansion while the other was used for microglia proliferation and isolation. Five days following the generation of the GFP+ cultures, WT cultures were generated from the SEZ of neonatal mice as described above. Confluent primary SEZ cultures were trypsinized and divided into three fractions. Fraction [1] was supplemented with GFP SEZ microglia; fraction [2] was supplemented with GFP CTX microglia; and fraction [3] served as an unsupplemented control. Samples of each fraction ($9x10^4$ cells plus $1x10^4$ isolated microglia) were plated in quadruplicate on P-Orn coated glass coverslips (surface area: 3.8cm2), as well as a TC25 plate for subsequent expansion, ($2.5x10^5$ cells plus $5x10^4$ microglia). One well was fixed 24 hours after plating and processed for immunohistochemical analysis to determine the number of donor (CD11b+/GFP+) and host (CD11b+/GFP-) microglia present in the cultures. Confluent passage 1 cultures were further expanded using the above design while the confluent cultures in the 12-well TC plates were processed for immunohistochemical analysis to determine the number of B-III tubulin+ neurons present in naïve and supplemented cultures.

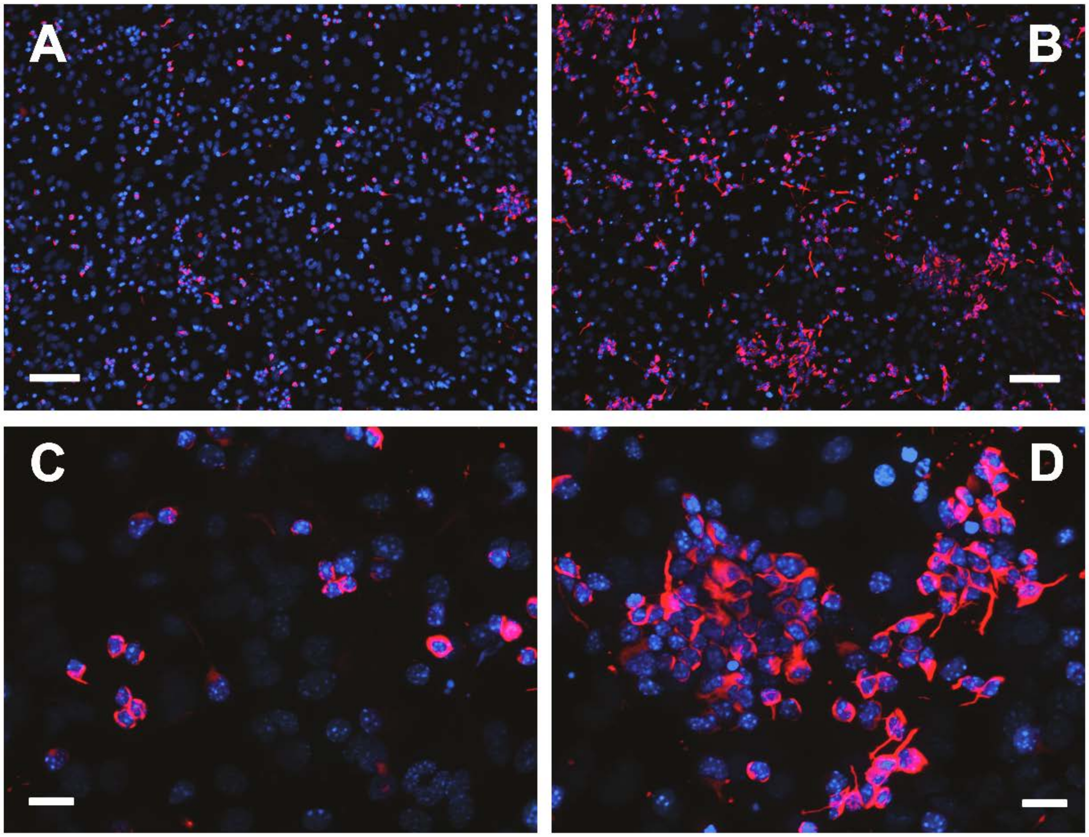

Supplemental Figure 8: Inducible neurogenesis in adherent SEZ-derived cultures. SEZ cultures (passage 2) contain a small number of B-III tubulin positive neurons (A, and C, red) sparsely dispersed throughout the culture. By 24 hours after induction (withdrawal of mitogens and serum) the number of neurons is substantially increased, with numerous clusters of neuroblasts apparent (B and D, red). Scale bar in A and B = 100um. Scale bar in C and D = 50um. Nuclei stained with DAPI (blue) in all images.

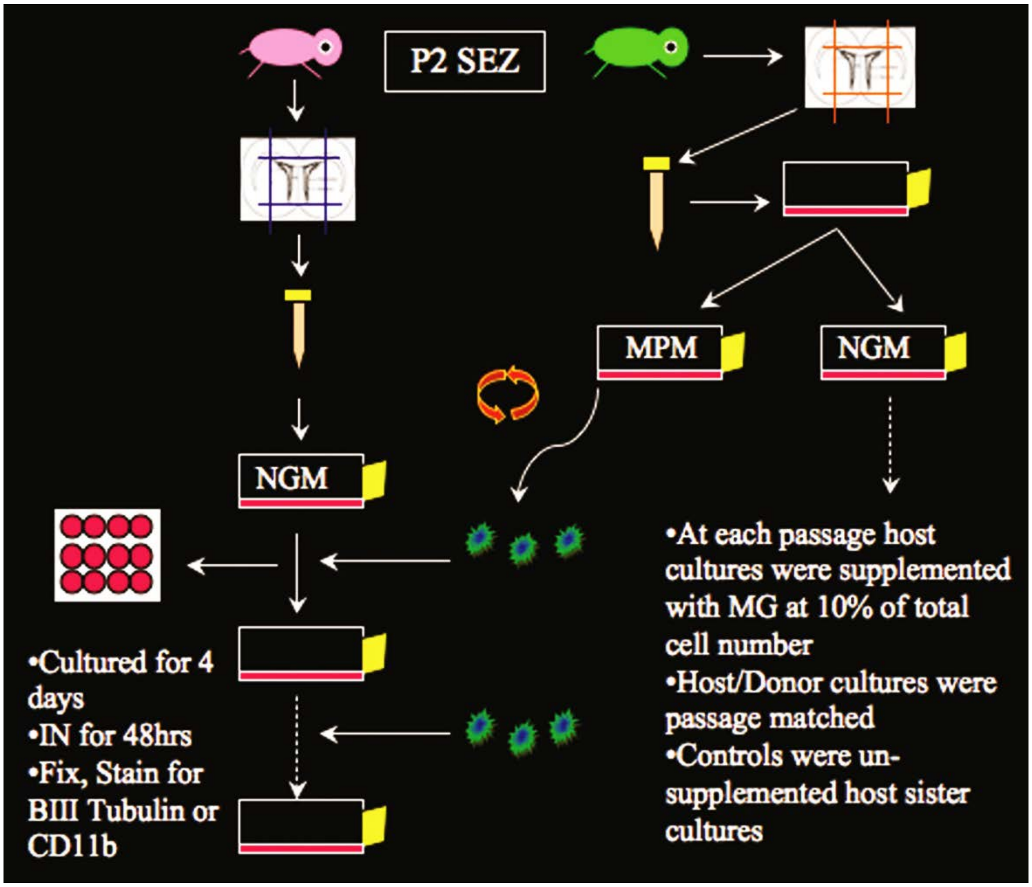

Supplemental Figure 9: Schema for preserving inducible neurogenesis by repeated supplementation with SEZ microglia. Adherent SEZ cultures were derived from GFP+ and WT neonatal mice as described above. The GFP+ culture was used to produce "donor" microglia, and was initiated 3 days prior to the "host" WT cultures in order to allow time for the expansion and isolation of donor microglia. When confluent primary adherent host cultures were passaged, donor microglia were added at 10% of the total host cell number. Aliquots of the cell mixtures ($9x10^4$ host cells plus $1x10^4$ isolated donor microglia) were plated in quadruplicate on P-Orn coated glass coverslips (surface area: 3.8cm2). Additional aliquots were plated in T-25 flasks for continued expansion, ($4.5x10^5$ host cells + $5x10^4$ donor microglia). Confluent passage 1 cultures were further expanded by repeating this protocol (out to passage 3) while triplicate confluent cultures on the glass coverslips were subjected to the IN protocol described below. Immunolabeling was performed to determine the relative number of neurons and microglia (both host and donor derived) present in naïve and supplemented cultures.
